# An Investigation in Sub-Millimeter Channel Fabrication by the Non-Aqueous Electrolyte Jet Machining of Zr-Based Bulk Metallic Glasses

**DOI:** 10.3390/mi14122232

**Published:** 2023-12-12

**Authors:** Cheng Guo, Aixing Zhou, Jingwen He, Huapan Xiao, Duo Li

**Affiliations:** 1 College of Mechatronics and Control Engineering, Shenzhen University, Shenzhen 518060, China; 2310295105@email.szu.edu.cn (A.Z.); hejingwen2021@email.szu.edu.cn (J.H.); 2 State Key Laboratory of Ultra-Precision Machining Technology, Department of Industrial and Systems Engineering, The Hongkong Polytechnic University, Hung Hom, Hong Kong, China; xhp0698@126.com; 3 Center for Precision Engineering, Harbin Institute of Technology, Harbin 150001, China; liduo@hit.edu.cn

**Keywords:** EJM, Zr-Based Bulk Metallic Glasses (BMGs), non-aqueous electrolyte, channel fabrication

## Abstract

Zr-based bulk metallic glasses (BMGs) have many unique properties. Due to their excellent performance and manufacturing process, they have become a research focus in the material science community. Electrolyte Jet Machining (EJM) is a non-contact electrochemical processing method with high surface integrity and high material removal rate (MRR). In this research, the sub-millimeter channels fabricated by EJM on Zr-based BMGs have been studied to explore the dissolution mechanisms and surface integrity under different scanning rates and voltages. The results show that, with other machining parameters holding constant, an increase in voltage leads to a substantial enhancement in both the depth and width of the channels machined on Zr-based BMGs. Notably, the influence of voltage on the depth of the channels is particularly pronounced. Additionally, an escalation in scanning rate correlates with a decrease in channel depth, with minimal variation in channel width. This study indicates that alcohol-based EJM is an effective method to fabricate sub-millimeter channels and modulate structures on Zr-based BMGs.

## 1. Introduction

Bulk Metallic Glasses (BMGs) are multi-component alloys that form an amorphous structure during the solidification process. The technical interest in these materials stems from their unique properties, which often surpass those of traditional structural materials [1]. Due to the presence of an amorphous structure and the exceptional performance of non-crystalline materials, BMGs demonstrate distinctive cutting characteristics during the diamond turning process. These characteristics include, but are not limited to, chip formation, cutting forces, tool wear, oxidation, and crystallization. In contrast to the drawbacks, such as low processing efficiency and severe tool wear, associated with laser machining and micro-electrical discharge machining, BMGs uniquely exploit their advantages in the microfabrication of amorphous alloys [2]. BMGs exhibit outstanding mechanical, physical, and chemical properties. To fully unleash the application potential of large BMGs, it is essential to establish appropriate processing techniques [3]. For instance, researchers like Lin et al. employed two types of nanosecond-pulsed laser systems for the microfabrication of Mg-based BMGs [4]. Xie et al. employed micro-milling techniques for processing BMGs. The research findings indicate that, with appropriate mechanical machining methods, achieving ideal geometric precision and surface quality on BMGs’ components is feasible [5]. Chen et al. utilized electrical discharge machining techniques to process BMGs. The study results revealed that BMG material is more effectively removed during the electrical discharge machining process, leading to a higher Material Removal Rate (MRR) [6].

Zr-based Bulk Metallic Glasses (BMGs) are emerging materials, prepared under special conditions. They demonstrate promising prospects for a wide range of applications [7]. Based on their distinctive microstructure, BMGs exhibit a variety of unique features, including exceptional magnetic properties, resistance to corrosion, durability against wear, hardness, and toughness [8], while, due to the excellent mechanical characteristics of Zr-based BMGs, employing traditional mechanical methods for material removal proves challenging. Differently to conventional mechanical methods, Electrolyte Jet Machining (EJM) is a kind of non-contact chemically removed process that concurrently demonstrates exceptional MRR and surface integrity.

As an efficient removal process, EJM enjoys a good reputation. Ao et al. used three different nozzle-moving speeds of 6 μm/s, 4 μm/s and 2 μm/s to fabricate channels for SiC-particle reinforced aluminum matrix composites (SiCp/Al) under selected process parameters. The results showed that although different nozzle-moving speeds were used, channels with acceptable shape accuracy could be successfully machined on SiCp/Al using EJM. The depth of the channels machined using nozzle-movement speeds of 6 μm/s, 4 μm/s, and 2 μm/s were 80 μm, 117 μm, and 135 μm, respectively [9]. Liu et al. adjusted the moving rate of the nozzle to 25 μm/s, controlled the gap between electrodes to 0.6 mm, and set the flow rate of electrolytes to 2.1 L/min with a voltage of 24 V. A typical channel pattern was fabricated on Ti1023 titanium alloy by EJM. It was found that the rough structure caused by stray corrosion was usually formed at the edge of the machining channel [10]. Chen et al. found that in jet mode, the duty cycle of current also had a significant impact on the machining of micro channels. Compared with other duty cycles, the duty cycle of 20% (10 V and 15 V) was conducive to reducing the size standard deviation and roughness of the micro channel, and the micro channel with a depth of less than 100 μm could be generated. On the contrary, when the voltage was increased to more than 20 V, the results showed that a micro channel depth greater than 100 μm could be formed. Increasing the number of reciprocating motion was conducive to improving the dimensional uniformity and roughness of the micro channel [11]. Hung et al. conducted EJM experiments on SUS304 stainless steel by controlling whether or not mixed gas was added to produce micro-scale channels. The efficiency levels of the two processing techniques were compared. The results showed that the mixing of gas and electrolytes can improve the machining efficiency and reduce the conductive area between the electrolyte and the workpiece [12]. Wang, X.D. et al. found that the blade’s stray corrosion could be significantly reduced by adjusting the shape of the jet, and established the necessary conditions for completely eliminating stray corrosion. On this basis, the channel with a sharp edge and smooth surface was processed through the double-pass process, and a deep channel with sharp edge was obtained through the multi-pass milling process with a downward cathode feed [13]. Yahyavi Zanjani et al. highlighted the importance of current density measurements in the control of jet electrolytic machining processes. They found that the current density was very sensitive to changes in the working gap. For a single channel, the current density changed significantly if the preset working gap was changed at a constant machining voltage. For a single channel, the depth of the channel was linearly related to the current density, and the surface roughness decreased first and then increased with the increase in the current density [14]. Mitchell-Smith et al. found that ultrasonic assistance could partially remove the passivation film formed, thereby improving the overall performance. They processed the Ti-6Al-4V alloy by ultrasonic-assisted electrolyte jet processing, and found that the passivation layer could be broken down by ultrasonic assistance, thereby improving the aspect ratio of the channel [15]. Wang et al. adjusted the nozzle-injection angle to −15° in the experiment, and found that the channel had a smooth silver surface. Compared with the nozzles with other jet angles, the channel processed by nozzles with a −15° jet angle had the largest MRR, flat bottom zone width, processing depth, and minimum taper [16]. The mechanical properties of the tungsten carbide alloy make it challenging to process traditionally. Therefore, Martin et al. machined channels on tungsten carbide alloys by electrochemical machining with a continuous free jet and pulsed current, and the results showed that a certain surface ripple degree could be machined on the tungsten carbide alloy by using parallel line cutting with pulsed-current EJM. When the wavelength corresponded to the lateral distance of a single channel, the defined wavelength could be machined by adjusting the line spacing [17]. Luo et al. proposed the arrangement of tubular electrodes with appropriate inter-tubular spacing, known as tube electrodes in a row, in EJM. They successfully employed this approach to process multiple channels on the workpiece [18]. Zhao et al. observed that, during the electrochemical milling of micro channels, the impact of current density was significantly greater than that of the electrode gap and the nozzle scanning rate. Consequently, they devised corresponding strategies for adjusting the machining parameters [19]. Using EJM, Kai et al. perforated a post-metallic sheet, employing a nozzle with a diameter of 210 μm. They successfully cut through a 3 mm thick stainless steel sheet, achieving a cut width ranging from 220 μm to 230 μm [20].

EJM can also be combined with other technologies. Wu et al. have developed a non-contact machining process known as Mask Electrolyte Jet Machining (MEJM) by combining high-resolution lithography with the greater flexibility of EJM, which can be utilized for creating various microstructures on the workpiece surface [21]. Zhang et al. observed that the MMR was lower when employing horizontal EJM compared to its vertical counterpart [22]. Huang et al. introduced ultrasonic waves into the electrolyte during the machining process, addressing the limitations of EJM [23]. Ho et al. proposed that employing visual-sensing methods would be an effective way to observe the penetration state of electrochemical discharge drilling in real-time [24]. Zhang et al. introduced a composite machining approach, combining EJM with Laser Beam Machining (JECM-LBM), effectively leveraging the advantages of both machining methods [25]. The introduction of laser assistance effectively enhanced the MRR and reduced tapering [26].

In this study, EJM has been employed to investigate the electrochemical milling of channels on BMGs in NaCl-Ethylene-Glycol (EG) electrolyte with a concentration of 1 mol/L. The water-based electrolytes often formed oxide layers during the machining process, hindering the uniform dissolution of the electrolytes and consequently reducing machining precision. On the other hand, the use of organic solvent electrolytes, such as NaCl-EG electrolyte, helped to avoid the formation of oxide layers throughout the entire EJM process. This enhanced the overall machining performance of EJM compared to water-based electrolytes [27]. This research primarily explores the influence of the voltage and the nozzle scanning rate on the dissolution mechanism and surface integrity of Zr-based BMGs, focusing on aspects such as the width, depth, and surface morphology of the machined channels. The experimental results indicate the significant impact of the voltage and the nozzle scanning rate on the sample morphology, particularly on the width and depth of the channels. To achieve better processing efficiency and quality, it is crucial to judiciously select the parameters for jet electrochemical machining.

## 2. Materials and Methods

**Figure 1:**
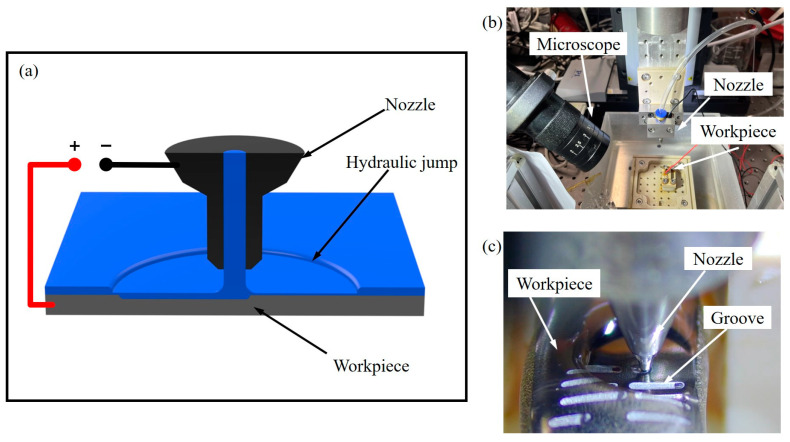
Experimental device schematic and physical pictures. (**a**) Illustration of EJM principle; (**b**) physical device; (**c**) workpiece process.

The working principle of the EJM system and experimental setup diagram have been illustrated in Figure 1. The basic idea was to eject the electrolyte at a set flow rate from a moving nozzle with an inner diameter of 200 μm, generating a circular hydraulic jump on the workpiece. The conductive liquid column, composed of the electrolyte, exhibited significantly higher current density at the center compared to the surrounding areas after applying electrical voltage. Thanks to the hydraulic jump, the electrolyte film diffused along the workpiece surface, and the current density could be focused under the nozzle. The electrolyte supply system employed a high-performance liquid chromatography pump, and the pump output flow rate was set to 60 mL/min for all experiments; however, influenced by the viscosity of the electrolyte, measurements indicate that the actual flow rate was 50 mL/min.

In Figure 1b, the nozzle is mounted on a three-axis moving platform with a precision of up to 0.1 μm. The workpiece to be processed was positioned directly beneath the nozzle’s outlet, and an initial gap of 200 μm was set to meet the hydraulic demand. The workpiece material was a high-performance Zr-based BMG, Vit1 (Zr_41.2_Ti_13.8_Cu_12.5_Ni_10.0_Be_22.5_), which had been polished with #7000 waterproof sandpaper. To minimize the formation of passive films, the 1 mol/L NaCl-EG electrolyte was used as the electrolyte. Cl¯ is highly invasive and typically does not induce passivation. Furthermore, the solvent, ethylene glycol, was non-aqueous, and its ability to generate oxygen was weak. Even in the presence of a passivation film, it remained quite thin, and high-flow electrolytes swiftly flushed this passivation film from the machining surface. The temperature of NaCl-EG electrolyte was 25 °C and the electrolyte flow rate was quite rapid, calculated to reach 26.5 m/s. The influence of flow rate on morphology primarily lay in controlling the convection and diffusion rates of electrolytic products. When the flow rate was high, these mass transfer processes tended to stabilize. In the machining process, fluctuations in electrolyte current density led to corresponding variations in electrolyte conductivity, showing a positive correlation between the two. For the 1 mol/L NaCl-EG electrolyte, its conductivity fell within the range of 0.12 S/m to 0.21 S/m [28]. The experimental power supply utilized in the experiment consisted of a signal generator and a power amplifier. The voltage and current signals during experiments were sampled using isolated probes. The experimental process was divided into multiple parts, on the basis of controlling a single variable. Both direct currents and pulse currents with a frequency of 5 kHz were applied to investigate the effects of different applied voltages and moving speeds on channel machining performance.

Through the analysis of the processed cross-sectional profiles and morphology, laser scanning confocal microscopy (LSCM) was employed to analyze the cross-sectional profiles (depth and width of the channel), while scanning electron microscopy (SEM) was used to observe the overall concave shape of the workpiece and the occurrence of stray corrosion.

## 3. Results and Discussions

In this section, the effects of voltage and scanning rate on the machining properties of channels are mainly characterized by comparing the morphology, depth and width. The scale bar in the measurement pictures below is 50 μm.

### 3.1. The Effect of Voltage

The voltage was adjusted from 60 V to 140 V. The duty cycle was controlled at 50%, and the effective processing time of the workpiece was 15 s. In addition, in order to investigate the influence of the nozzle scanning rate on the machining performance of the workpiece, two groups of parameters were compared; the first group adjusted the nozzle scanning rate to 50 μm/s, and the second group adjusted the nozzle scanning rate to 70 μm/s. The relevant parameters are listed in Table 1.

#### 3.1.1. At the Nozzle Scanning Rate of 50 μm/s

**Figure 2:**
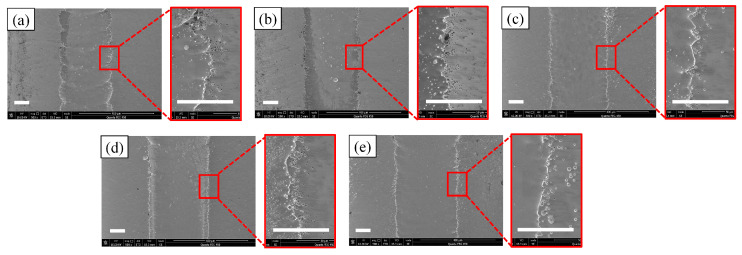
SEM morphologies at different voltages (50 μm/s). (**a**) 60 V; (**b**) 80 V; (**c**) 100 V; (**d**) 120 V; (**e**) 140 V.

**Figure 3:**
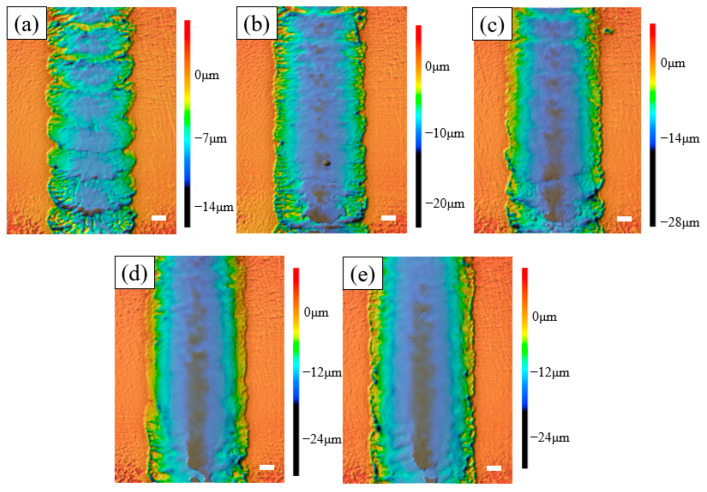
The depth cloud map at different voltages (50 μm/s). (**a**) 60 V; (**b**) 80 V; (**c**) 100 V; (**d**) 120 V; (**e**) 140 V.

**Figure 4:**
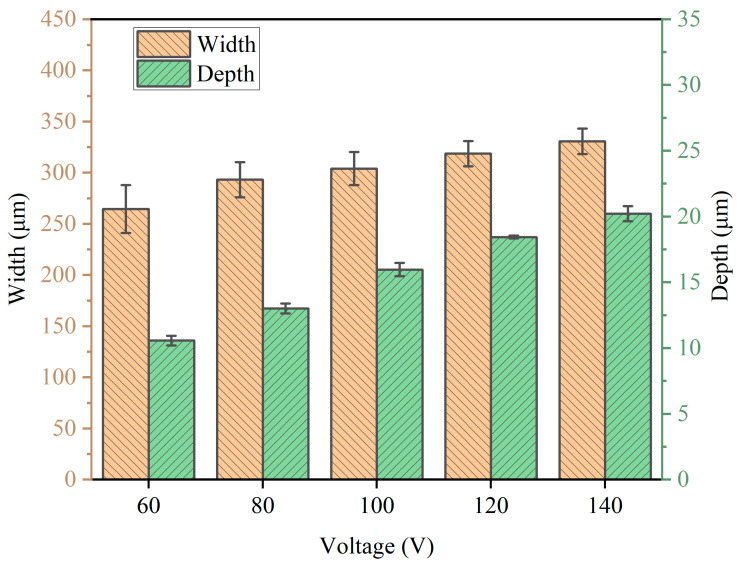
Diameter and depth at different voltages (50 μm/s).

The corresponding SEM diagrams under these processing parameters are shown in Figure 2. It can be observed from the figure that after the workpiece was machined out of the channel, the edge of the channel was uneven, and when the applied voltage was 60 V, the edge of the channel was the most uneven. Stray corrosion also appeared at both ends of the channel. When the voltage was 140 V, the corrosion of the edge reached the maximum, but at this time, the machining surface quality of the channel was significantly improved. Waves also appeared on the processed surface of the channel, but this phenomenon was improved with the gradual increase in voltage; when the voltage reached 140 V, the wave could be observed to almost disappear. In addition, there were micro holes in the stray corrosion on both sides of the channel, and with the increase in voltage, these micro holes also gradually increased. The depth cloud map of each channel is analyzed in Figure 3. It is shown that when the applied voltage gradually increased, the depth of the channel also increased. When the applied voltage was 60 V, its depth ranged from 7 μm to 10 μm. When the voltage reached 80 V, the depth of the channel was between 10 μm and 13 μm. It also be observed that the channel surface processing accuracy was improved and surface waviness decreased. When the voltage reached 100 V, the depth of the channel was between 14 and 16 μm, and the surface wave almost disappeared. When the voltage reached 120 V, it could be found that the stray corrosion area on both sides of the channel increased, with the depth ranging from 16 μm to 18 μm. When the voltage reached 140 V, the maximum depth reached about 19 μm, and the stray corrosion area on both sides of the channel also reached the maximum. To obtain a deeper channel, it was not advisable to greatly increase the voltage, because it would lead to more serious stray corrosion and uneven phenomena on both sides of the channel. The variation trend of the maximum depth and width of the channel with the increase in voltage is expressed in the bar chart, as shown in Figure 4. It could be obviously found that with the gradual increase in voltage, the maximum depth and width of the channel increased. The width change was very significant with the increase in voltage. When the voltage was only 60 V, the width of the channel was about 264 μm. When the voltage reached 140 V, the channel appeared to be at the maximum depth (ca. 19 μm) and the maximum width (ca. 330 μm).

#### 3.1.2. At the Nozzle Scanning Rate of 70 μm/s

**Figure 5:**
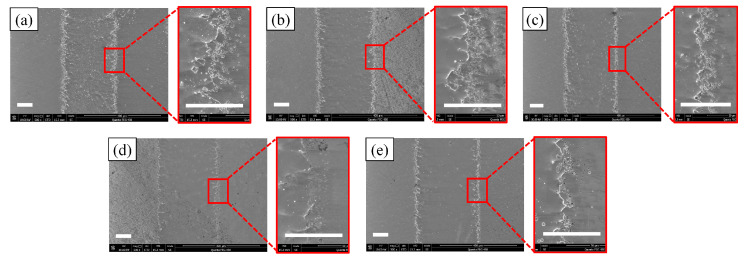
SEM morphologies at different voltages (70 μm/s). (**a**) 60 V; (**b**) 80 V; (**c**) 100 V; (**d**) 120 V; (**e**) 140 V.

**Figure 6:**
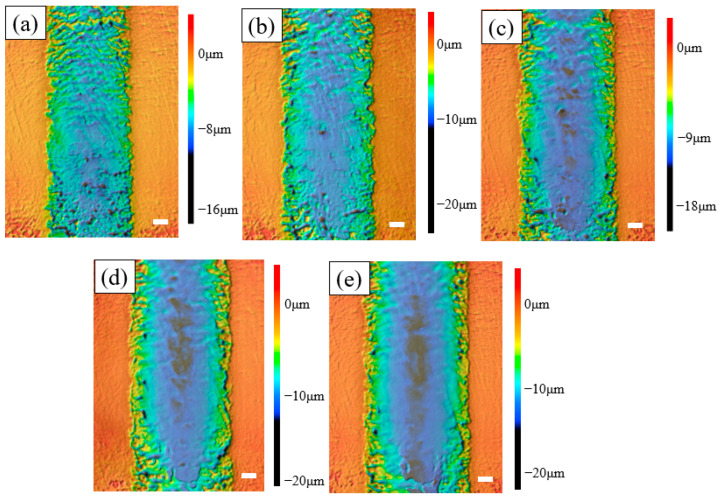
The depth cloud map at different voltages (70 μm/s). (**a**) 60 V; (**b**) 80 V; (**c**) 100 V; (**d**) 120 V; (**e**) 140 V.

**Figure 7:**
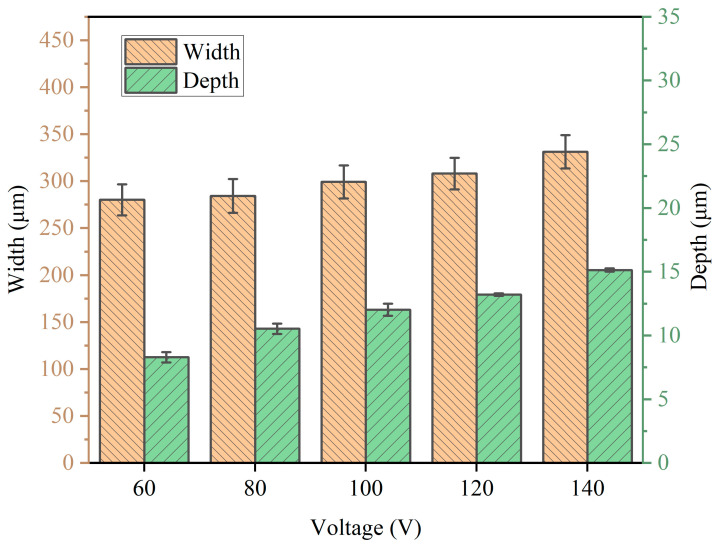
Diameter and depth at different voltage (70 μm/s).

In this group, the remaining working parameters were kept unchanged, as above. The nozzle scanning rate was increased to 70 μm/s, and the working voltage was gradually increased from 60 V to 140 V. The SEM diagram of the channel processed by the workpiece under corresponding working parameters is shown in Figure 5. It could be found that noticeable waves were on the surface of the channel when the voltage was 60 V; at this voltage, the current density was 19.1 A/cm^2^. However, compared with the previous group, it had been greatly improved, considering the fact that when the nozzle scanning rate was increased, the timing for the electrolyte liquid column on the processed surface of the workpiece was shortened, and the overall machining was faster, so that the fluctuation degree of the channel surface could be reduced. In addition, in this group, stray corrosion and micropores could be found on both sides of the channel. The stray corrosion was the most serious and the micropores were the largest when the voltage reached 140 V and the current density was 44.6 A/cm^2^. At the same time, both uneven sides of the channel indicated that simply increasing the nozzle scanning rate could not eliminate fluctuation of the channel. The depth cloud map of each channel is analyzed in Figure 6. Compared with the previous group, when the voltage was 60 V, the channel surface waviness was greatly improved, with the channel depth ranging from 6 μm to 8 μm. When the voltage was 80 V, the current density reached 25.5 A/cm^2^ and the channel depth was distributed between 8 μm and 10 μm. When the voltage was 100 V, the channel depth increased a little. When the voltage was 120 V, the channel surface accuracy had been greatly improved. When the voltage reached 140 V, the channel surface machining accuracy was the highest and the depth was ca. 15 μm. With the voltage increase, the channel depth also gradually increased. Figure 6 shows that the channel depth of the channel increased with the gradual increase in the applied voltage. In Figure 7, it is shown that the maximum depth and maximum width of channels after machining changed with the increase in voltage when the nozzle scanning rate was 70 μm/s. At this rate, the maximum depth and maximum width of the channel gradually increased. When the voltage was 60 V, the maximum width of the channel was 280 μm and the maximum depth was 8 μm. When the voltage reached 140 V, the maximum width of the channel was 331 μm. Compared with the case under the scanning rate of 50 μm/s, it could be found that the maximum depth of channel machining was not much different, but the maximum width of channel machining was significantly different. When the voltage was 60 V, the maximum depth of the channel was 10 μm for the case with the nozzle scanning rate of 50 μm/s, while the maximum depth of the channel group was only 8 μm for the case with the nozzle scanning rate of 70 μm/s. When the voltage reached 140 V, the difference between the two groups was more obvious, and the maximum depth of the channel in the first group was 20 μm. In contrast, the maximum depth of the second set of channels was only 15 μm. The reason for this is that the faster the nozzle moved, the shorter the residence time of the electrolyte liquid column washing on the workpiece, and the shorter the electrochemical machining time; so, in the two groups of different voltages, the maximum depth of the channel with the nozzle scanning rate of 70 μm/s was less than that under the scanning rate of 50 μm/s.

### 3.2. The Effect of the Scanning Rate

#### 3.2.1. Morphologies

**Figure 8:**
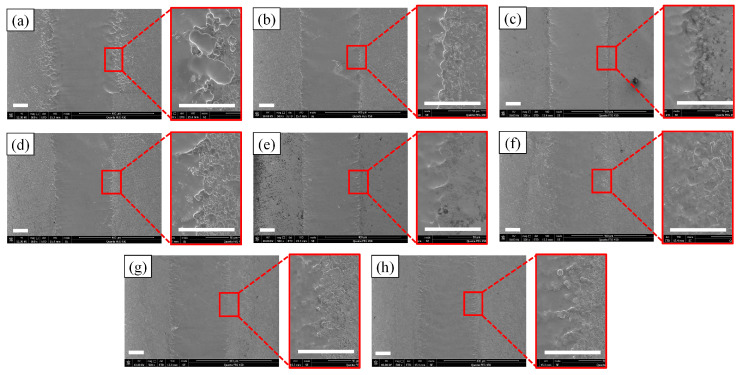
SEM morphologies at different scanning rates. (**a**) 30 μm/s; (**b**) 40 μm/s; (**c**) 50 μm/s; (**d**) 60 μm/s; (**e**) 70 μm/s; (**f**) 80 μm/s; (**g**) 90 μm/s; (**h**) 100 μm/s.

In this part, the influence of the nozzle scanning rate on the workpiece channel was mainly explored, and the scanning rate was increased by 10 μm/s for each group starting from 30 μm/s, with other parameters remaining unchanged (Table 2). The results are shown in Figure 8. When the scanning rate was 30 μm/s, the boundaries on both sides of the channel were extremely uneven, and the surrounding stray corrosion was more obvious; because the scanning rate was low, there was a longer electrolytic machining time, resulting in irregular boundaries and the serious stray corrosion of the channel. However, with the increase in the scanning rate, this phenomenon was improved; it can be seen that when the scanning rate reached 100 μm/s, the machining accuracy of the channel was greatly improved, the uneven phenomenon of the boundary was also improved, and the stray corrosion on both sides of the channel was also reduced. In addition, with the increase in scanning speed, the waviness of the channel also decreased.

#### 3.2.2. Geometric Characterization

**Figure 9:**
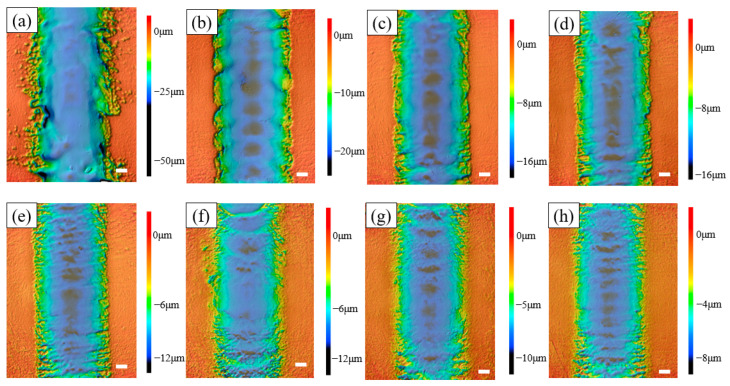
The depth cloud map at different scanning rates. (**a**) 30 μm/s; (**b**) 40 μm/s; (**c**) 50 μm/s; (**d**) 60 μm/s; (**e**) 70 μm/s; (**f**) 80 μm/s; (**g**) 90 μm/s; (**h**) 100 μm/s.

In the depth cloud map (Figure 9), it can be intuitively observed that when the scanning rate was 30 μm/s, the channel processing accuracy was very poor, the stray corrosion on both sides was obvious, and the two sides were very uneven. When utilizing EJM for grooving, the central region of the jet stream experienced a heightened current density. Employing a scanning technique through the nozzle within this central area produced a U-shaped groove with a superior surface quality. Nevertheless, beyond this specified region, there was a significant drop in current density. As one moves farther from the central axis of the groove, the current density diminishes progressively. This leads to the exposure of the lateral edges of the groove to a lower current density, resulting in material susceptibility to selective or scattered corrosion [13]. The depth of the channel was ca. 26 μm, which also shows that in order to obtain a deeper processing depth of the channel, only reducing the nozzle scanning rate was not desirable. The precision of the channels produced by this processing were bad. When the scanning rate was 40 μm/s, it could be found that the stray corrosion phenomenon on both sides of the channel had been greatly improved, and the depth was ca. 20 μm. When the scanning rate gradually increased, it could be found that the two sides were increasingly flush. However, when the scanning rate further increased, such as to 90 μm/s and 100 μm/s, it was found that parts outside the channel were also corroded.

#### 3.2.3. Cross-Sectional Shape Analysis

**Figure 10:**
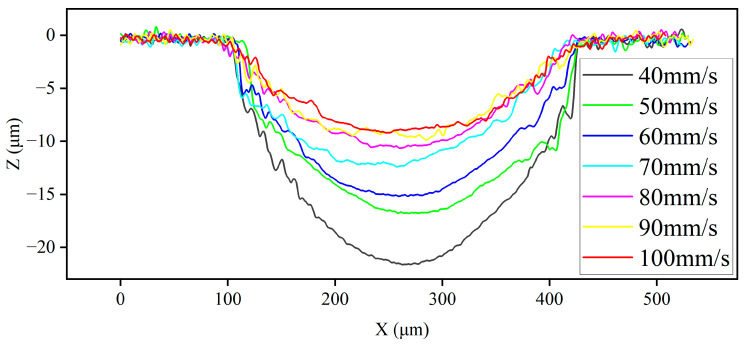
Cross-section profiles under different scanning rates.

**Figure 11:**
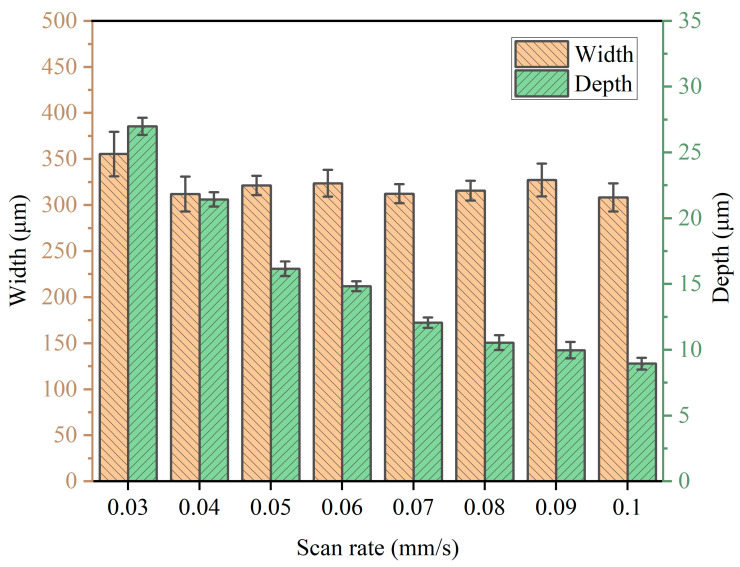
Diameter and depth at different scanning rates.

As shown in Figure 10, significant scattered corrosion was observed at the edges of the channel when the nozzle scanning rate was 30 μm/s. With the scanning rate gradually increasing, it could be found that the maximum machining depth of the channel was gradually reduced because the higher scanning rate of the nozzle led to a shorter processing time for the electrolyte column to scour the channel surface, so less material was removed and the maximum depth of the channel was smaller. In addition, it could be found that the surface quality difference of the channel center was not obvious, but in the end area, the curve was non-uniform. The farther away from the machining center, the lower the current density, which caused the uneven removal of material. Further statistics of the maximum depth and maximum width of each group of channels are shown in Figure 11. There was no significant difference in the maximum width of the channels. When the scanning rate was 30 μm/s, the maximum width of the channel was 355 μm, and the maximum width of the other groups of channels was above 300 μm. The maximum depth of channel machining was 27 μm when the scanning rate was 30 μm/s. When the scanning rate was 100 μm/s, the maximum depth of the channel was only 9 μm. This shows that with the increase in scanning rate, the maximum depth of the channel decreased, and when the scanning rate was within a low level, the maximum depth difference of the channel processing changed more obviously. When the scanning rate reached a certain value, this difference reduced.

## 4. Conclusions

In this research, the channel fabrication of Zr-based bulk metallic glasses (BMGs) has been achieved through electrolyte jet machining. Given that water-based salt electrolytes tend to induce the formation of passive films on the workpiece surface, NaCl-Ethylene Glycol (EG) has been employed as the electrolyte. The processed channels have been characterized using Scanning Electron Microscope (SEM) and Laser Scanning Confocal Microscopy (LSCM) to explore the feasibility of this electrochemical machining technique. The study primarily compares and analyzes the effects of voltage and nozzle scanning rate. The main conclusions are summarized as follows:

(1) With other processing parameters held constant, increasing voltage significantly influences the depth of the workpiece channels. As voltage rises, the depth of the channels gradually increases, although the width also experiences a slight augmentation. Nevertheless, excessive voltage should be avoided in the pursuit of greater depth, as this may lead to more pronounced stray corrosion on the sides of the channels. For the impact of the nozzle scanning rate on the channels, the experimental findings reveal that with an increase in scanning rate, the depth of the channels decreases, while the width shows minimal variation. This is primarily attributed to the diminishing effective time of the electrolyte column on the workpiece surface with the rising scanning rate, resulting in a reduction in material removal rate.
(2) The EG electrolyte can effectively remove Zr-based BMGs materials. This suggests that the EG electrolyte is adept at preparing multi-component alloys like Zr-based BMGs. In the experiment, while achieving a smooth surface in the machined channels, there still remained significant scattered corrosion and flow marks on both sides of the channels.
(3) Under the appropriate processing parameters, such as a voltage of 100 V and a nozzle scanning rate of 80 μm/s, a satisfactory surface quality for the channels can be achieved. Furthermore, there is a notable reduction in stray corrosion on both sides of the channels.

## Figures and Tables

**Table 1:** Experimental parameters of voltage groups.

Voltage (V)	t_cyc_ (μs)	Duty Cycle (%)	Electrode Diameter (μm)	Gap (μm)	Flow Rate (mL/min)
60, 80, 100, 120, 140	200	50	200	200	50

**Table 2:** Experimental parameters of scanning rate groups.

Voltage (V)	t_cyc_ (μs)	Duty Cycle (%)	Scanning Rate (μm/s)	Gap (μm)	Flow Rate (mL/min)
100	200	50	30, 40, 50, 60, 70, 80, 90, 100	200	50

## Data Availability

The data presented in this study is openly available.
